# Effect of Different Early Weaning Diets on Survival, Growth, and Digestive Ontogeny of *Channa striatus* (Bloch, 1793) Larvae

**DOI:** 10.3390/ani14192838

**Published:** 2024-10-01

**Authors:** Pandi Kalaiselvan, Amit Ranjan, Mir Ishfaq Nazir, Eswaran Suresh, Albin Jemila Thangarani, Kavitha Malarvizhi

**Affiliations:** 1 Institute of Fisheries Post Graduate Studies, Tamil Nadu Dr. J. Jayalalithaa Fisheries University, Vaniyanchavadi 603103, India; kalaiprs1641@gmail.com (P.K.);; 2 Directorate of Incubation and Vocational Training in Aquaculture, Tamil Nadu Dr. J. Jayalalithaa Fisheries University, Muttukadu 603112, India; 3 Faculty of Fisheries, Sher-e-Kashmir University of Agricultural Sciences and Technology, Shalimar, Srinagar 190025, India

**Keywords:** *Channa striatus*, digestive enzymes, larval ontogeny, weaning, protease supplementation

## Abstract

**Simple Summary:**

*Channa striatus* (striped murrel) is a valuable freshwater fish known for its taste and medicinal properties. Despite the advances in breeding technology, larval rearing remains a major challenge for producing high-quality seeds for commercial production. A key issue is the lack of a scientifically validated weaning protocol, resulting in high mortality during early developmental stages. After feeding different early weaning diets over 29 days, we have found that larvae fed with *Artemia* nauplii increased the overall larval performance during the initial exogenous feeding phase. However, constraints due to feeding solely with *Artemia* nauplii necessitate weaning with an artificial micro diet. By 12 dph, the performance of larvae fed with *Artemia* nauplii and co-feed diet (*Artemia* nauplii and formulated diet) showed comparable results, and by 20 dph, the larvae fed with protease supplemented formulated micro diet showed better performance compared to other treatments. Therefore, this study gives an insight for standardizing weaning protocol for *C. striatus* larvae based on the effects of different early weaning diets, which positively enhances survival and growth of the *Channa striatus* larvae.

**Abstract:**

The present study was carried out to evaluate the survival, growth, and digestive ontogeny of *C. striatus* larvae fed with different experimental diets from 4 days post-hatch (dph) to 32 dph at three-day intervals. A total of 24,000 larvae, with 1600 larvae per tank in triplicate and an initial mean weight of 0.64 ± 0.01 mg at 4 days post hatch (dph) were subjected to five different early weaning diets, namely *Artemia* nauplii (T1), co-feed diet comprising *Artemia* nauplii and formulated micro diet (T2), formulated micro diet (T3), formulated micro diet with protease supplementation (T4), and a commercial diet (T5). All the early weaning diets significantly affected the survival, growth, and ontogeny of the digestive system. Initially at 8 dph, *C. striatus* fed with T1 showed better survival and growth performance compared to other treatments. By 12 dph, the larvae fed with T1 and T2 showed similar results in terms of survival and growth performance, outperforming other treatments. However, the larvae fed with T2 and T4 outperformed T1 in survival and growth performance at 16 dph. By 24–32 dph, the larvae fed with all treatments met the basic nutritional needs for survival, with T4 fed larvae showing better growth compared to other treatments. At the end of the trial, cumulative mortality was lowest in larvae fed with T1 and highest in the larvae fed with T3 and T5. Similarly, the larvae fed with T4 showed significantly higher weight gain, specific growth rate (SGR), and average daily growth (ADG), while T1 fed larvae exhibited better feed conversion ratio (FCR) and protein efficiency ratio (PER). The enzyme activity fluctuated throughout the experimental duration. Lavae fed with T1 and T2 showed higher enzyme activities initially. However, T4 fed larvae showed higher trypsin and chymotrypsin specific activity at 16 dph along with well-developed intestinal folds with dense microvilli, higher pepsin-specific activity at 20 dph onwards with fully developed gastric glands and thicker gastric mucosal epithelium, and higher amylase and lipase activity at 16 dph with large and prominent zymogen granules in the exocrine pancreas. Peaking at 4 dph, the activity of protein metabolic enzymes (AST and ALT) sharply declined at 8 dph and increased until 32 dph. Larvae fed with T1 showed higher AST and ALT activity along with increased lipid deposits, followed by those fed with T2 and the larvae fed with T4 showing higher activity without fat accumulation but significantly lower than those fed T1 and T2. From the present research findings, it is recommended to initiate weaning for *Channa striatus* larvae with *Artemia* nauplii (from 4 dph to 8 dph) followed by a co-feeding regime (*Artemia* nauplii and formulated diet) between 9 and 16 dph and transition to protease-supplemented micro diet (T4) from 17 dph onwards.

## 1. Introduction

In aquaculture, *Channa striatus* (striped murrel) is a valuable freshwater species with significant importance. It is considered a crucial food fish due to its air-breathing ability, superior meat quality, and medicinal properties [1,2,3]. Although there was a slight increase in striped murrel production in 2020, the significant decrease in global production was noteworthy [4]. According to FAO [5], production dropped to 80,626 tons, down from 90,719 tons in 2020. Therefore, it is crucial to enhance the fish production through aquaculture to fulfill the future needs, which depends on the steady supply of good quality seeds [3,6]. In India, striped murrel consumption primarily depends on wild collection (capture fisheries) because the production of high-good quality fingerlings is limited in terms of both quantity and quality due to high mortality during the larval phase [7,8]. Several factors contribute to the high mortality affecting the commercial production of striped murrel, including its cannibalistic nature, poor management of feed and broodstock, and lack of effective nutritional plans and rearing protocols [9,10]. While advancements have been made in induced seed production in striped murrel, the larval rearing remains highly challenging, particularly due to the lack of scientifically validated weaning protocol [3,11]. When establishing an ideal and effective weaning strategy for fish larvae, it is crucial to consider several factors, including determining the right time to begin larval weaning, optimal feeding frequency, and ration based on the digestive capabilities on particular stages of larval morphogenesis [12,13]. Cahu and Zambonino-infante [14] and Lazo et al. [15] have reported that the optimal initial larval feeding varies among species and is primarily tied to the maturation of the gastrointestinal system. Improper age or diet during the weaning process can harm the digestive system and cause chronic stress leading to progressive starvation, which in turn delays or impairs the growth, health, and overall physiological function of fish larvae. The success of weaning is also determined by the timing of occurrence and the secretion of digestive enzymes [16].

Digestive enzymes play a crucial role in efficiently utilizing ingested feed. However, the development of the digestive system and its associated glands are genetically pre-programmed and influenced by various factors, such as environmental parameters, type of feed, and its nutritional composition [17]. Numerous investigations have demonstrated the importance of digestive enzymes as indicators for determining the development of gastrointestinal system and dietary health of fish larvae [16,18]. The quantification of digestive enzymes during the ontogenical development of striped murrel will be a classical approach in understanding digestive capabilities and timing of the physiological development of the digestive system that provides a baseline in standardizing the weaning protocol based on the nutritional needs at each phase of larval development. Being a carnivorous fish, the larvae require live feed during the initial exogenous nutritional phase of which *Artemia* nauplii are the best choice for striped murrel compared to other live feeds because it has a balanced nutritional profile, easily acceptable and readily digested during the early developmental stages, and larvae lack completely developed digestive system [19,20,21]. Although *Artemia* nauplii are beneficial as an initial feed for larvae, it might not always contain all the essential nutrients to meet the complete dietary requirements of the larvae in subsequent stages for optimal growth and development [22]. In addition, the extensive usage of *Artemia* nauplii has led to a high demand for its cysts, driving up the price in the market [23]. Moreover, the production of live feed requires a considerable amount of hatchery space and exclusive setups, making it a labor-intensive activity and costly process. Therefore, replacing live feed with a formulated micro diet will ensure proper nutrition to developing larvae and reduce overall production cost [23,24]. However, the use of a micro diet soon after hatching often leads to reduced survival and impaired growth performance of fish larvae. This may be attributed to decreased digestibility, unbalanced nutrient composition, and reduced feed palatability, shape, color, and size of the feed and nutrient leaching associated with micro diets [13,14,18,23,25]. Recently, functional micro diets are blooming up to improve the growth performance and survival of fish larvae, one of which is supplementing it with protease enzymes to enhance the digestibility and early development of the digestive system [21].

Several studies have concluded that co-feeding, a combination of live feed and micro diet, has advantages in alleviating issues such as poor acceptability and digestibility associated with micro diets at the early stages of development. It also enhances the achievement of early weaning with formulated dry feed [18,23,26,27]. The two accepted reasons can explain this success. First, live prey contains unidentified nutritional elements that trigger pancreatic secretions that provoke the endocrine responses beneficial for the development of the digestive system [3]. Second, the visual and chemical cues from live prey facilitate the ingestion of micro diet, which positively impacts the larval growth [12,18]. Live feed provides a variety of nutritional factors that not only supply necessary nutrients for the micro diet but also support gut development by stimulating the secretion of pancreatic enzymes, thus preparing the larvae for early weaning [13,25]. In recent years, the supplementation of protease enzymes has been a growing interest, showing the potential for enhancing nutrient digestibility that subsequently increases the growth and health performance of fish larvae [3,21,28]. During larval rearing, the need for a protease-supplemented diet is crucial in effectively breaking down the complex nutrients and in efficient absorption, resulting in better growth and survival rate [3,29]. With the above background, the present study was designed to investigate the effects of different early weaning diets from 4 days post-hatch (dph) to 32 dph on survival, growth, and activity of digestive and metabolic enzymes to study the ontogenical development of the digestive tract and establish a standardized weaning protocol for *C. striatus* larvae.

## 2. Materials and Methods

### 2.1. Experimental Design and Diet Formulation

The experimental diets used in this study were divided into different treatments, consisting of live feed, a formulated micro diet (with or without protease supplementation), and a commercial diet, as shown in Table 1. In Treatment-1 (T1), the larvae were fed with Brine shrimp nauplii (BSN) at the instar stage I as live feed. In Treatment2 (T2) and Treatment-3 (T3), larvae were fed with a formulated micro diet containing crude protein, CP—52% of dry weight and crude lipid, and CL—12% of dry weight, but without protease enzyme. This micro diet was formulated using fish meal (FM), fish protein hydrolysate (FPH), soy protein concentrate (SPC), squid meal (SM), shrimp head meal (SHM), fish oil, sunflower oil, soy lecithin, wheat flour, carboxy methyl cellulose, vitamin premix, mineral premix, vitamin E, vitamin C, L-tryptophan, and protease enzyme as mentioned in Table 2. In Treatment-2, the larvae were weaned on a diet consisting of both live and formulated feed, which were fed in an alternative feeding schedule. Specifically, BSN instar I was administered at 08.00 h, followed by the formulated diet at 12.00 h. The same feeding schedule was repeated at 16.00 h and 20.00 h, alternating between BSN instar I and the formulated diet. In Treatment-4 (T4), larvae were fed with a formulated micro diet supplemented with protease enzymes derived from *Bacillus* sp. (200,000 u/g, pH 6–12) with adjustments in the wheat flour content. In treatment-5 (T5), larvae were fed with a commercial diet (0.3 mm pellets, Nutrila™—Growel Feeds Pvt, Ltd. Singarayapalem, Andhra Pradesh, India) containing CP—52% of dry weight and CL—12% of dry weight as mentioned in Table 3.

### 2.2. Diet Preparation

The dry powdered ingredients, including fish meal, squid meal, shrimp head meal, wheat flour, and carboxy methyl cellulose were sieved through an 80-mesh screen. Except fish protein hydrolysate, soy protein concentrate, feed additives, and oil, all the ingredients were weighed and mixed according to the ingredient composition of the experimental diets as shown in Table 2. Following the attainment of a homogenous mixture of dry ingredients, it is made into a dough by gradually blending it with water to achieve a proper consistency. Subsequently, the resultant dough was wrapped in a wet cloth and transferred to the autoclave for cooking, where it undergoes steaming for 20 min. Post-steaming, the other ingredients, namely fish protein hydrolysate, soy protein concentrate, sunflower oil, fish oil, soy lecithin, vitamin premix, mineral premix, vitamin C, vitamin E, and L-tryptophan, were thoroughly mixed with the cooled dough. A hand pelletizer was used to obtain a uniform pellet size of 2 mm. The experimental diets were then dried at room temperature in a well-ventilated room. The pellet size was further reduced using a pulverizer, resulting in powdered feed. The obtained powder was sieved through a 0.3 mm mesh screen procured from a local hypermarket (Vollum flour sifter of size 12” diameter × 2.5” height) suitable for the larvae’s mouth size and then stored in labeled, moisture-free, air-tight containers at room temperature. In the case of larvae fed with T4, the prepared micro diet is manually top-coated with a protease enzyme dissolved in distilled water, dried at ambient temperature, and then fed to the larvae. For the larvae fed with T1 and T2 (co-fed with formulated micro diet), hatched *Artemia* nauplii instar I (PRO 80, ©Ocean Star International, INC. Snowville, UT, USA) was used as live feed as mentioned in Table 3. During the hatching process, the eggs were incubated in artificial saltwater (28–30 ppt at 28–30 °C). Vigorous aeration was provided by connecting four air stones with the same aerator used for larval rearing, and constant illumination (2000 lux) was provided using a 100 W Crompton bulb for an efficient and high hatching rate. During harvesting, the aerator was turned off after 24 h to siphon out the accumulated BSN instar I towards the light.

### 2.3. Experimental Animal Source and Husbandry

The *Channa striatus* larvae of an average weight of 0.64 ± 0.01 mg and average total length of 0.56 ± 0.03 mm for the experimental trial were procured from CR Aqua Tec Pvt Ltd., Hatchery, Udipalya, Bangalore, India on the 4 dph, i.e., soon after the absorption of the yolk sac. The larvae were carefully shifted in the double-layer polyethylene (PE) bag from the procurement site to the indoor experimental site of the hatchery. After acclimatization, a total of 24,000 larvae were stocked, with one thousand six hundred (1600 nos.) larvae per replicates, across three replicates per treatment. The experimental trial was conducted in 100 L rectangular glass tanks (50 × 40 × 50 cm) and maintained with the water volume of 70 L. The larvae were fed with the respective experimental weaning diets (viz. T1–T5) manually from 4 dph to 32 dph and at 08.00, 12.00, 16.00, and 20.00 h (IST) until satiation. The larvae in the T2 were fed as indicated previously in Section 2.1. The feeding was stopped soon after the cessation of foraging behavior and the assembly of larvae at the corners and the bottom of the tank. The leftover feed, fecal waste, and dead larvae were siphoned out during water exchange (50% of total volume) daily before the onset of the first feeding at 08.00 h. Each experimental tank was connected to an air stone diffuser linked to the aerator (10 W) to ensure optimal food circulation and oxygenation. Optimal water quality was maintained consistently throughout the experimental feeding trial. The temperature ranged between 26 and 29 °C, pH levels were observed within 7.5–7.7, dissolved oxygen content varied from 6 to 8 mg/L, nitrite and nitrate level was ranged between 0–0.5 and 0–5 mg/L, and total ammonia nitrogen levels were observed to be between 0.05 and 0.10 mg/L. All the water quality parameters were analyzed using commercially available water test kits manufactured by R.S. Fish Farm, Kolkata, India.

### 2.4. Sampling of Larvae

A total of 300 numbers of 4 dph larvae were collected from the same batch of experimental larvae at the procurement site before starting the experimental trial for conducting initial growth measurements and digestive enzyme assays. After the commencement of the feeding trial, larvae were sampled from each tank after overnight fasting to collect the larval samples for estimating growth and enzyme assays (trypsin, chymotrypsin, pepsin, lipase, amylase, total protease, aspartate aminotransaminase, and alanine aminotransaminase). Before the morning feeding, the overnight starved larval samples were randomly collected at three-day intervals, i.e., 8 dph (n = 20), 12 dph (n = 20), 16 dph (n = 15), 20 dph (n = 10), 24 dph (n = 10), 28 dph (n = 10), and 32 dph (n = 7) from each experimental tank. The variation in the number of larvae sampled at different sampling points is due to the need to maintain sufficient biomass for accurate biochemical enzyme quantification. As the larvae grow over time, their biomass increases (wet weight). Hence, only fewer larvae were sampled to provide enough larval samples for enzyme analysis at later stages. The collected larval samples were sacrificed by immersing them in ice-cold water and subsequently rinsed with distilled water. Daily mortality was counted to estimate the survival rate and cumulative mortality rate. At the end of the feeding trail, total biomass of the larvae was used to calculate final growth measurements including average daily growth (ADG), mean weight gain (MWG), feed conversion ratio (FCR), specific growth rate (SGR), and protein efficiency ratio (PER).

### 2.5. Proximate Composition

Proximate composition was estimated for the feed ingredients and experimental diets according to AOAC [30]. Moisture content was calculated by drying the samples at 100 ± 2 °C to constant weight. Crude protein (CP) was estimated by the Kjeldhal method (N × 6.25) after acid digestion (Foss™, Digestor™ 2508 from Foss Tecator line, Foss India Pvt Ltd., Mumbai, India) followed by distillation (Foss™, Kjeltec™ 8100, Foss India Pvt Ltd., Mumbai, India) and titration. Crude lipid (CL) was calculated by extracting the samples with petroleum ether as an extracting solvent using the Soxhlet method (SOCS Plus™, Pelican equipment, Chennai, India). The ash content was estimated by placing appropriate samples in the porcelain crucible and kept inside the muffle furnace for incineration at 550 °C for 6 h.

### 2.6. Sample Preparation and Enzyme Assay

The collected samples from each rearing tanks were pooled in Eppendorf tubes (5 mL) and homogenized with ice-cold 50 mM Tris HCl buffer (pH 7.5) using electronic motor homogenizer (Remi RQ 124A/D, Remi Elektrotechnik Ltd., Vasai, India). After homogenization, the samples were centrifuged at 5000× *g* at 4 °C for 10 min and the supernatant was collected and stored at −20 °C. The activities of digestive enzymes were analyzed with the standard methods. In brief, the activities of trypsin and chymotrypsin were estimated by the casein digestion method, according to the method of Kunitz [31]. For trypsin, the reaction mixture containing 0.1 M phosphate buffer (pH 7.6), 1% casein as substrate, and tissue homogenate was incubated for 20 min at 37 °C. After incubation and filtration by Whatman filter paper No.1, the absorbance of the sample mixture was recorded at 280 nm. For the estimation of chymotrypsin activity, the reaction mixture containing 0.1 M borate buffer with CaCl2 at pH of 8, 1% casein as substrate, and tissue homogenate was incubated for 20 min at 37 °C. The absorbance was measured at 280 nm. One unit of enzyme activity was defined as the amount of micromole of tyrosine released at 37 °C. The activity of pepsin was quantified by using the method of Worthington [32]. The enzyme reaction mixture containing substrate 2% hemoglobin in 0.3 N HCl, pH and tissue homogenate was incubated for 10 min at 37 °C. Later, the enzymatic reaction was terminated using adding 5% trichloroacetic acid. The supernatants from assay tubes were collected after centrifuging the reaction mixture at 4000 g at 4 °C for 6 min. One unit of enzyme activity was defined as the amount of tyrosine released per minute per milliliter at 37 °C. Estimation of total protease activity was conducted by the casein digestion method of Drapeau [33]. The enzymatic reaction mixture containing 1% casein in 0.05 M Tris-phosphate buffer (pH 7.8) was added with tissue homogenate and kept for incubation for 10 min. Then, the reaction was stopped by adding 10% TCA followed by filtration using Whatman filter paper No.1. The absorbance was measured at 280 nm. One unit of enzyme activity was defined as the amount of enzyme needed to release soluble acid fragments equivalent to 1 mmol at 280 nm per minute at 37 °C and pH 7.8. The activity of the amylase enzyme was evaluated by using a starch solution dissolved in phosphate buffer (pH 6.9) as substrate [34]. 0.1 mL of enzyme extract was incubated with substrate solution at 95 °C for 4 min. Later, 2 mL of dinitro-salicylic acid (DNS) was added, kept in a boiling water bath for 5 min, and then allowed to cool. After diluting with 5 mL of distilled water, the activity was measured by reading the absorbance at 540 nm. Using maltose as standard, the specific amylase activity was expressed as a micromole of maltose released from starch per minute at 37 °C. The activity of lipase was determined by the Cherry and Crandall [35] method using olive oil emulsion as a substrate. The reaction mixture was prepared with 1 mL of sample homogenate, 0.5 mL of phosphate buffer solution (pH 7.0), 2 mL of olive oil emulsion, and distilled water and incubated at 37 °C. After 24 h, the reaction mixture was added with 3 mL of 95% alcohol and 2 drops of phenolphthalein and titrated against 0.05 N NaOH until a permanent pink color appeared. The activity of protein metabolic enzymes, including AST and ALT in the larval samples were measured by using a SGOT and SGPT kit (Erba Diagnostics, Mannheim, Germany) as per the protocol given in the kit. The activity was measured by reading the absorbance at 340 nm and expressed as U/L.

With bovine albumin serum as a standard, the Bradford method (1976) was used to quantify the soluble protein in the enzyme extract using an ELISA Microplate Reader (BioTek Epoch 2, Agilent Technologies, Chennai, India) at 595 nm. The absorbance was recorded using Cary 60 UV-Vis Spectrophotometer, Agilent Technologies, Santa Clara, CA, USA and expressed as specific enzyme activity (U/mg protein and U/L).

### 2.7. Histomorphological Analysis of Digestive System Development

To prepare the specimens for histology, two larval specimens were randomly selected at each sampling point from each replicate along with the enzyme sample collection. The sampled larvae were killed by immersing them in ice-cold water and rinsed with distilled water. Later, the samples were fixed with 10% neutral buffered formalin (NBF) in respective Eppendorf tubes (5 mL) labeled according to the treatments. The collected samples were replaced by NBF every 24 h and preserved in 70% ethanol. The fixed individual larval samples were sent to Whizbang Bioresearch Pvt Ltd., Chennai, India, for histological processing and slide preparation. The preserved specimens are initially dehydrated with a series of ethanol washes (70%, 95%, and 100%). Then, it was infiltrated at a melting point of 56–57 °C for 1 h using paraffin wax. Later, casting or blocking was performed by embedding it in the paraffin using embedding rings, and the blocks were placed at 4 °C for 15 min for solidification. A serial sagittal section of 7 µm was cut using a rotatory microtome, set in a water bath (45 °C), and placed on scintillating slides. Then, the slides were allowed to dry overnight in the oven at 37 °C before being stained by a standard histological stain (Hematoxylin-eosin, H-E). The stained slides were examined using a digital compound microscope (Optscopes E5 series, Germany). Microphotographs of the sections were taken with the 5.1-megapixel ultra-cam fixed microscope adaptor attached in the same microscope. The effect of different weaning diets on the ontogeny of the gastrointestinal system during early developmental stages was interpreted according to Lazo et al. [15] and Paray et al. [36].

### 2.8. Calculations and Statistical Analyses

The calculation formulas were as follows:

1. Average daily growth (ADG) = (Final weight (mg)−Initial weight (mg)/Trial period in days)
2. Mean weight gain (MWG) = Final mean weight (mg)/Initial mean weight (mg)
3. Feed conversion ratio (FCR) = Dry matter feed intake (g)/Wet weight gain (g)
4. Protein efficiency ratio (PER) = Wet weight gain (g)/Dry weight of total protein fed (g)
5. Specific growth rate (SGR) = (Ln of wet weight at the end of the trial−Ln of wet weight at the beginning of the trial)/Trial period in days × 100
6. Survival = ((Live number of larvae left in the tank−number of sampled larvae)/initial number of larvae) × 100
7. Cumulative mortality (CM) = (Total number of dead larvae/Initial number of larvae) × 100

All data were assessed for normality and homogeneity of variance using the Shapiro–Wilk test and Levene’s test prior to performing one-way analysis of variance (ANOVA) with IBM SPSS, version 27. A post hoc multiple comparison test using Ducan’s test was then performed to identify significant differences between the experimental groups. Statistically significant differences or relationships were defined as having a probability of occurring by chance less than 5% (*p* <0.05). The results were presented in the format of mean ± standard error.

## 3. Results

### 3.1. Larval Survival and Cumulative Mortality RATE

**Figure 1:**
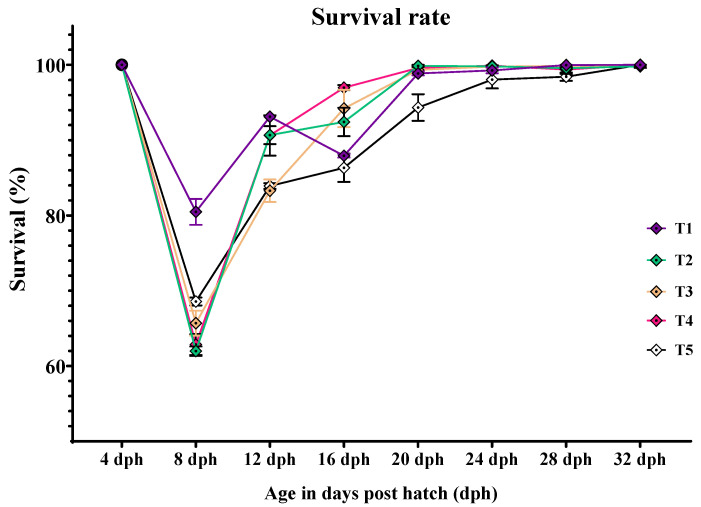
Survival and cumulative mortality rate of *C. striatus* larvae fed different experimental diets.

The initial stocking of 1600 larvae were stocked in each replicate of the treatments at 4 dph. The survival rate and cumulative mortality of larvae reared from 4 dph to 32 dph is presented in Table 4 and graphically depicted in Figure 1. The survival rate of *C. striatus* larvae had a significant difference (*p* < 0.05) with the different treatments from 8 dph to 20 dph. At 8 dph, highest (80.479 ± 1.71%) and lowest (61.979 ± 0.62%) survival was found in the larvae fed with T1 and T2 diet. At 12 dph, there is no significant difference (*p* > 0.05) in the survival rate of the larvae fed with T1, T2, and T4 diets, but significantly (*p* < 0.05) higher than those fed with T3 and T5 diets. The larvae fed with T4 diet showed the highest (96.973 ± 0.33%) survival rate, followed by the larvae fed with T3 (94.206 ± 2.47%) and T2 (92.414 ± 1.89%), and the lowest (86.328 ± 1.88%) was obtained in the larvae fed with T5 diet at 16 dph. At 20 dph, the survival rate of the larvae fed T1, T2, T3, and T4 diets were significantly higher than T5 diet but not significant among each other. The survival rate of *C. striatus* larvae did not vary significantly (*p* > 0.05) with the different treatments from 24 to 32 dph. At the end of the feeding trial, the cumulative mortality rate of *C. striatus* larvae varied significantly (*p* < 0.05) with the different treatments. The larvae fed with T5 (51.708 ± 2.01%) and T3 (48.313 ± 1.04%) showed significantly (*p* < 0.05) higher cumulative mortality rate, followed by T4 (43.208 ± 1.63%) and T2 (47.292 ± 0.47%), and the lowest (34.875 ± 1.65%) was obtained in the larvae fed T1 diet.

### 3.2. Growth Performance

The *C. striatus* larvae, with a mean initial body weight of 0.64 ± 0.01 mg were stocked for a feeding trial with the different treatments. Different weaning diets significantly (*p* < 0.05) affected the growth performance of larvae from 4 dph to 32 dph. The growth data is presented in Table 5. At 8 dph, the larvae fed with T1 (24.896 ± 0.14 mg) showed the highest significant difference (*p* < 0.05) in mean weight, followed by those fed with T2 (23.020 ± 0.17 mg), which is significantly higher than T4 (21.016 ± 0.20 mg), while the lowest mean weight was observed in the larvae fed with T3 (17.863 ± 0.29 mg) and T5 (17.896 ± 0.11 mg). At 12 dph, the larvae fed T1 (47.136 ± 0.20 mg) and T2 (47.110 ± 0.28 mg) showed the highest mean weight followed by those fed T4 (42.066 ± 0.14), which was significantly higher than T3 (32.813 ± 0.42) and T5 (32.833 ± 0.18). At 16 dph, the larvae fed with T2 (71.620 ± 0.40 mg) and T4 (71.600 ± 0.40) showed the highest mean weight, followed by the larvae fed with T1 (68.533 ± 0.38), while the lowest mean weight was observed in the larvae fed with T3 (65.023 ± 0.15 mg) and T5 (64.556 ± 0.37 mg). At 20 dph, the mean weight of the larvae fed with T4 (128.156 ± 0.18 mg) showed highest significant difference (*p* < 0.05) compared to other treatment groups. From 24 to 32 dph, the larvae fed with T4 showed the highest significant difference (*p* < 0.05) in the mean weight compared to other treatment groups.

At the end of the feeding trial, the larvae fed with T4 (8.456 ± 0.007 mg) showed significantly (*p* < 0.05) higher average daily growth, followed by those fed with T3 (8.151 ± 0.006 mg) and T5 (8.124 ± 0.17 mg), and the lowest was obtained in the larvae fed with T1 (6.688 ± 0.01 mg). Similarly, the larvae fed T4 (245.230 ± 0.20 mg) showed significantly (*p* < 0.05) higher mean weight gain, followed by T3 (236.407 ± 0.17 mg) and T5 (235.607 ± 0.52) and the lowest was obtained in the larvae fed with T1 (193.966 ± 0.51 mg). The significantly (*p* < 0.05) higher FCR was obtained in the larvae fed with T5 (5.658 ± 0.27) and T3 (5.347 ± 0.20), followed by T4 (4.53 ± 0.03) and T2 (3.529 ± 0.03), and the lowest was obtained in the larvae fed with T1 (1.889 ± 0.17). The significantly (*p* < 0.05) higher PER was found in the larvae fed with T1 (1.015 ± 0.08), followed by T2 (0.534 ± 0.004) and T4 (0.416 ± 0.002), while the lowest was obtained in the larvae fed with T5 (0.341 ± 0.01) and T3 (0.353 ± 0.01). The larvae fed with T4 (20.363 ± 0.002%) showed higher SGR, followed by T3 (20.236 ± 0.002%) and T5 (20.225 ± 0.007%), while the lowest was obtained in the larvae fed with T1 (19.609 ± 0.05%).

### 3.3. Ontogeny of Digestive Enzymes in Channa striatus Larvae Fed Different Experimental Diets

#### 3.3.1. Trypsin

**Figure 2:**
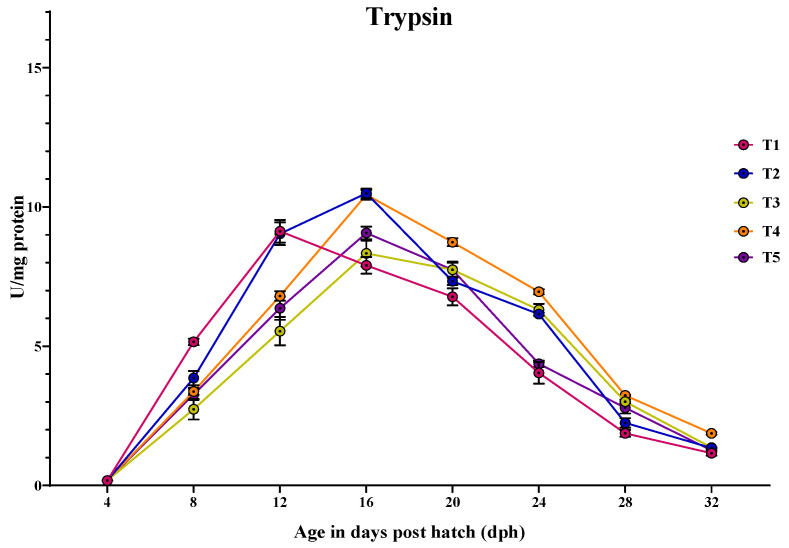
Ontogenical pattern of trypsin-specific activity of *C. striatus* fed different experimental diets.

Trypsin-specific activity was increased from 4 dph, peaking at 16 dph in all the treatments except the larvae fed with T1, where the peak occurred earlier at 12 dph, followed by a decline by 32 dph (Figure 2). At 4 dph, there was no significant difference (*p* > 0.05) obtained in the trypsin activity among the treatments. The trypsin activity varied significantly (*p* < 0.05) between the different treatments at all sampling points from 8 to 32 dph. At 8 dph, the larvae fed with T1 (5.155 ± 0.11 U/mg protein) showed the highest trypsin-specific activity, followed by those fed with T2 (3.854 ± 0.25 U/mg protein). T3 (2.736 ± 0.36 U/mg protein) had the lowest activity, while T4 (3.375 ± 0.12 U/mg protein) and T5 (3.270 ± 0.19 U/mg protein) showed intermediate activities, with no significant difference between them. At 12 dph, the larvae fed with T1 (9.125 ± 0.40 U/mg protein) and T2 (9.037 ± 0.40 U/mg protein) showed significantly (*p* < 0.05) higher trypsin activity compared to those fed with T4 (6.803 ± 0.17 U/mg protein), T5 (6.360 ± 0.41 U/mg protein), and T3 (5.542 ± 0.50 U/mg protein), which did not differ significantly from each other. Similarly, at 16 dph, the larvae fed with T2 (10.488 ± 0.17 U/mg protein) and T4 (10.437 ± 0.17 U/mg protein) showed significantly (*p* < 0.05) higher trypsin activity, followed by the larvae fed with T5 (9.065 ± 0.22 U/mg protein) and T3 (8.331 ± 0.46 U/mg protein), with the lowest activity obtained in the larvae fed with T1 (7.903 ± 0.30 U/mg protein). At 20 dph, the larvae fed with T4 (8.733 ± 0.14 U/mg protein) showed significantly (*p* < 0.05) higher trypsin activity compared to those larvae fed with T5 (7.745 ± 0.28 U/mg protein) and T3 (7.744 ± 0.24 U/mg protein), while the lowest activity was found in those larvae fed with T1 (6.775 ± 0.30 U/mg protein). At 24 dph, the larvae fed with T4 (6.953 ± 0.08 U/mg protein) showed significantly (*p* < 0.05) higher trypsin activity, followed by those fed with T3 (6.303 ± 0.21 U/mg protein) and T2 (6.153 ± 0.05 U/mg protein), while the lowest activity was obtained in the larvae fed with T5 (4.369 ± 0.11 U/mg protein) and T1 (4.042 ± 0.38 U/mg protein). Similarly, the larvae fed with T4 (3.26 ± 0.02 U/mg protein) showed significantly (*p* < 0.05) higher trypsin activity, followed by those fed with T3 (3.011 ± 0.06 U/mg protein) and T5 (2.791 ± 0.20 U/mg protein), with the larvae fed with T2 (2.247 ± 0.16 U/mg protein) and T1 (1.869 ± 0.12 U/mg protein) showing the lowest activity at 28 dph. At 32 dph, the larvae fed with T4 (1.87± 0.04 U/mg protein) showed significantly (*p* < 0.05) the highest trypsin activity compared to other treatments.

#### 3.3.2. Chymotrypsin

**Figure 3:**
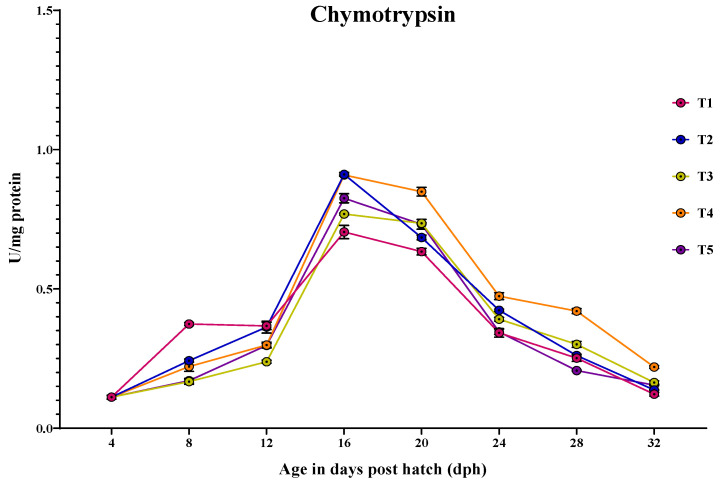
Ontogenical pattern of chymotrypsin-specific activity of *C. striatus* fed different experimental diets.

The ontogenical pattern of chymotrypsin-specific activity was similar to trypsin-specific activity peaking at 16 dph and decreased by 32 dph (Figure 3). At 4 dph, there was no significant difference (*p* > 0.05) in the chymotrypsin activity among the treatments. The chymotrypsin activity varied significantly (*p* < 0.05) between the different treatments at all sampling points from 8 to 32 dph. The larvae fed with T1 (0.374 ± 0.006 U/mg protein) showed significantly (*p* < 0.05) the highest chymotrypsin activity compared to other treatments at 8 dph. At 12 dph, the highest chymotrypsin activity was observed in the larvae fed with T1 (0.367 ± 0.01 U/mg protein) and T2 (0.362 ± 0.02 U/mg protein), which were significantly (*p* < 0.05) higher than those fed with T4 (0.298 ± 0.01 U/mg protein) and T5 (0.297 ± 0.007 U/mg protein), while the lowest activity was obtained in the larvae fed with T3 (0.238 ± 0.005 U/mg protein). At 16 dph, the larvae fed with T2 (0.911 ± 0.006 U/mg protein) and T4 (0.909 ± 0.002 U/mg protein) showed significantly (*p* < 0.05) highest chymotrypsin activity, followed by those fed with T5 (0.825 ± 0.01 U/mg protein) and T3 (0.768 ± 0.003 U/mg protein), while the lowest activity was found in the larvae fed with T1 (0.704 ± 0.02 U/mg protein). From 20 to 32 dph, the larvae fed with T4 consistently showed significantly (*p* < 0.05) the highest chymotrypsin activity compared to other treatments.

#### 3.3.3. Pepsin

**Figure 4:**
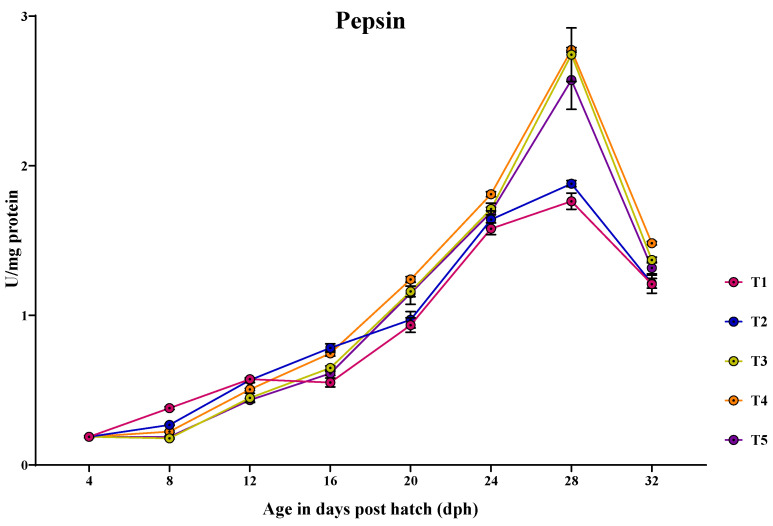
Ontogenical pattern of pepsin-specific activity of *C. striatus* fed different experimental diets.

Pepsin-specific activity was detected earlier at 4 dph, peaking at 20 dph and remained high until 32 dph (Figure 4). At 4 dph, there was no significant difference (*p* > 0.05) in the pepsin activity among the treatments. At 8 dph, the larvae fed with T1 (0.380 ± 0.01 U/mg protein) showed significantly (*p* < 0.05) highest pepsin activity compared to other treatments. At 12 dph, significantly (*p* < 0.05) highest pepsin activity was observed in the larvae fed with T1 (0.573 ± 0.002 U/mg protein) and T2 (0.567 ± 0.01 U/mg protein) than in those fed with T4 (0.505 ± 0.006 U/mg protein) and the lowest activity was found in the larvae fed with T5 (0.434 ± 0.01 U/mg protein) and T3 (0.448 ± 0.03 U/mg protein). At 16 dph, the larvae fed with T2 (0.782 ± 0.02 U/mg protein) and T4 (0.746 ± 0.01 U/mg protein) showed significantly (*p* < 0.05) highest pepsin activity, followed by the larvae fed with T3 (0.648 ± 0.01 U/mg protein), and the lowest activity was observed in those larvae fed with T1 (0.551 ± 0.02 U/mg protein). At 20 dph, the larvae fed with T4 (1.23 ± 0.01 U/mg protein), T3 (1.160 ± 0.03 U/mg protein), and T5 (1.148 ± 0.07 U/mg protein) showed significantly (*p* < 0.05) highest pepsin activity, which did not differ significantly from each other and the lowest activity was obtained in those larvae fed with T2 (0.970 ± 0.05 U/mg protein) and T1 (0.935 ± 0.04 U/mg protein). At 24 dph, the larvae fed with T4 (1.809 ± 0.01 U/mg protein) showed significantly (*p* < 0.05) higher pepsin activity, followed by the larvae fed with T3 (1.711 ± 0.03 U/mg protein) and T5 (1.689 ± 0.03 U/mg protein) and the lowest activity was obtained in the larvae fed with T2 (1.640 ± 0.05 U/mg protein) and T1 (1.579 ± 0.03 U/mg protein), which did not differ significantly from each other. At 28 dph, the larvae fed with T4 (2.773 ± 0.01 U/mg protein), followed by the larvae fed with T3 (2.742 ± 0.17 U/mg protein) and T5 (2.572 ± 0.19 U/mg protein) showed significantly (*p* < 0.05) highest pepsin activity, which did not differ significantly from each other and the lowest activity was obtained in those larvae fed with T2 (1.880 ± 0.02 U/mg protein) and T1 (1.762 ± 0.05 U/mg protein). At 32 dph, the larvae fed with T4 (1.482 ± 0.01 U/mg protein) showed significantly (*p* < 0.05) higher pepsin activity, compared to those fed with T3 (1.370 ± 0.01 U/mg protein) and the lowest activity was obtained in the larvae fed with T2 (1.214 ± 0.03 U/mg protein) and T1 (1.209 ± 0.06 U/mg protein).

#### 3.3.4. Total Protease

**Figure 5:**
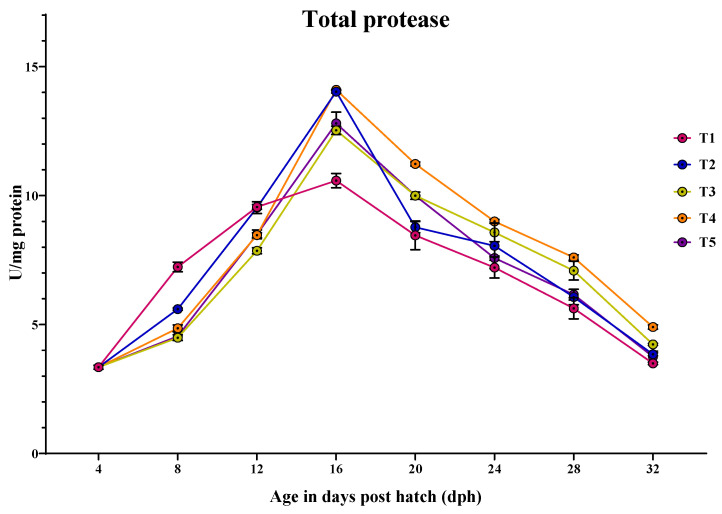
Ontogenical pattern of total protease activity of *C. striatus* fed different experimental diets.

Total protease activity was increased from 4 dph, reaching its peak at 16 dph and decreased towards 32 dph (Figure 5). At 4 dph, there was no significant difference (*p* > 0.05) in the total protease activity among the treatments. At 8 dph, the larvae fed with T1 (7.232 ± 0.18 U/mg protein) showed significantly (*p* < 0.05) highest total protease activity compared to the other treatments. At 12 dph, highest total protease activity was obtained in the larvae fed with T1 (9.557 ± 0.09 U/mg protein) and T2 (9.533 ± 0.23 U/mg protein), followed by those fed with T5 (8.495 ± 0.17 U/mg protein) and T4 (8.468 ± 0.08 U/mg protein) and the lowest activity was obtained in the larvae fed with T3 (7.858 ± 0.11 U/mg protein). At 16 dph, the larvae fed with T4 (14.098 ± 0.17 U/mg protein) and T2 (14.026 ± 0.04 U/mg protein) showed significantly (*p* < 0.05) highest total protease activity, followed by those fed with T5 (12.800 ± 0.43 U/mg protein) and T3 (12.532 ± 0.15 U/mg protein) and the lowest activity was obtained in the larvae fed with T1 (10.578 ± 0.27 U/mg protein). At 20 dph, the larvae fed with T4 (11.224 ± 0.07 U/mg protein) showed significantly (*p* < 0.05) highest total protease activity, followed by those fed with T3 (9.998 ± 0.01 U/mg protein) and T5 (9.994 ± 0.14 U/mg protein) and the lowest activity was obtained in the larvae fed with T2 (8.773 ± 0.217 U/mg protein) and T1 (8.458 ± 0.55 U/mg protein). At 24 dph, there was no significant (*p* > 0.05) difference was obtained among the treatments. At 28 dph, the larvae fed with T4 (7.602 ± 0.09 U/mg protein) showed significantly (*p* < 0.05) highest total protease activity, followed by those fed with T3 (7.091 ± 0.36 U/mg protein) and the lowest activity was obtained in the larvae fed with T2 (6.067 ± 0.29 U/mg protein) and T1 (5.627 ± 0.40 U/mg protein). At 32 dph, the larvae fed with T4 (4.902 ± 0.08 U/mg protein) showed significantly (*p* < 0.05) highest total protease activity, and the lowest activity was obtained in the larvae fed with T2 (3.846 ± 0.05 U/mg protein), T5 (3.750 ± 0.21 U/mg protein) and T1 (3.495 ± 0.04 U/mg protein), which did not differ significantly from each other.

#### 3.3.5. Lipase

**Figure 6:**
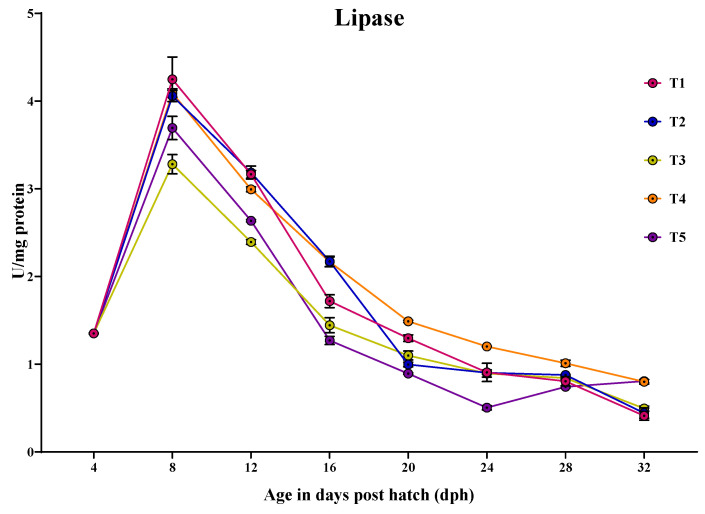
Ontogenical pattern of lipase-specific activity of *C. striatus* fed different experimental diets.

Lipase-specific activity was detected at 4 dph, peaking at 8 dph and then decreased towards 32 dph (Figure 6). At 4 dph, there was no significant difference (*p* > 0.05) in the lipase activity among the treatments. At 8 dph, the larvae fed with T1 (4.248 ± 0.25 U/mg protein) showed significantly (*p* < 0.05) higher lipase activity, followed by those fed with T4 (4.092 ± 0.04 U/mg protein) and T2 (4.058 ± 0.06 U/mg protein), with the lowest activity obtained in the larvae fed with T3 (3.280 ± 0.10 U/mg protein). At 12 dph, the larvae fed with T2 (3.185 ± 0.07 U/mg protein) and T1 (3.165 ± 0.04 U/mg protein) showed significantly (*p* < 0.05) the highest lipase activity and the lowest activity was obtained in the larvae fed with T3 (2.392 ± 0.02 U/mg protein). At 16 dph, the larvae fed with T2 (2.172 ± 0.05 U/mg protein) and T4 (2.166 ± 0.05 U/mg protein) showed significantly (*p* < 0.05) the highest lipase activity, followed by those fed with T1 (1.718 ± 0.07 U/mg protein) and the lowest activity was obtained in the larvae fed with T3 (1.444 ± 0.08 U/mg protein) and T5 (1.270 ± 0.04 U/mg protein). At 20 dph, the larvae fed with T4 (1.488 ± 0.01 U/mg protein) showed significantly (*p* < 0.05) higher lipase activity, with the lowest activity obtained in the larvae fed with T5 (0.893 ± 0.009 U/mg protein). At 24 dph, the larvae fed with T4 (1.201 ± 0.002 U/mg protein) showed significantly (*p* < 0.05) highest lipase activity, with the lowest obtained in the larvae Fed T5 (0.690 ± 0.01 U/mg protein). Similar, the larvae fed with T4 (1.011 ± 0.03 U/mg protein) showed highest lipase activity, followed by those fed with T2 (0.877 ± 0.006 U/mg protein) and T3 (0.841 ± 0.01 U/mg protein) and the lowest lipase activity was obtained in the larvae fed with T5 (0.741 ± 0.11 U/mg protein) at 28 dph. At 32 dph, the larvae fed with T5 (0.806 ± 0.01 U/mg protein) and T4 (0.799 ± 0.02 U/mg protein) showed significantly (*p* < 0.05) higher lipase activity compared to other treatments.

#### 3.3.6. Amylase

**Figure 7:**
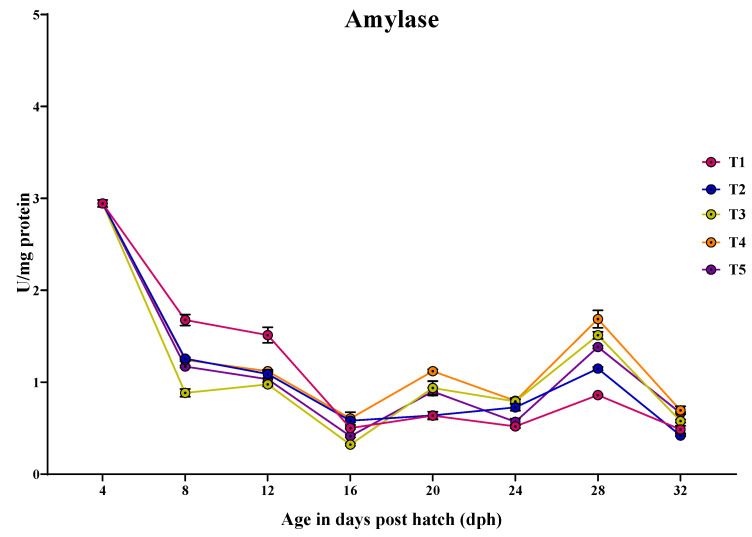
Ontogenical pattern of amylase-specific activity of *C. striatus* fed different experimental diets.

The specific activity of amylase showed a peak at 4 dph, followed by a drastic reduction with another peak at 28 dph in all treatments (Figure 7). At 4 dph, there was no significant difference (*p* > 0.05) in the amylase activity among the treatments. At 8 dph, the larvae fed with T1 (1.676 ± 0.05 U/mg protein) showed significantly (*p* < 0.05) the highest amylase activity compared to other treatments, which did not differ significantly from each other. Similarly, at 12 dph, the larvae fed with T1 (1.513 ± 0.08 U/mg protein) showed the highest activity compared to other treatments. At 16 dph, the larvae fed with T4 (0.605 ± 0.06 U/mg protein) and T2 (0.581 ± 0.01 U/mg protein) showed significantly (*p* < 0.05) higher amylase activity, followed by those fed with T1 (0.501 ± 0.01 U/mg protein) and T5 (0.413 ± 0.01 U/mg protein), and the lowest activity obtained in the larvae fed with T3 (0.321 ± 0.01 U/mg protein). At 20 dph, the larvae fed with T4 (1.120 ± 0.01 U/mg protein) showed significantly (*p* < 0.05) the highest amylase activity, followed by those fed with T3 (0.936 ± 0.07 U/mg protein) and T5 (0.899 ± 0.04 U/mg protein), and the lowest activity was obtained in the larvae fed with T1 (0.636 ± 0.04 U/mg protein) and T2 (0.639 ± 0.03 U/mg protein). At 24 dph, the larvae fed with T4 (0.798 ± 0.02 U/mg protein), T3 (0.791 ± 0.02 U/mg protein), and T2 (0.726 ± 0.03 U/mg protein) showed significantly (*p* < 0.05) the highest amylase activity, and the lowest activity was obtained in the larvae fed with T1 (0.519 ± 0.01 U/mg protein) and T5 (0.568 ± 0.03 U/mg protein). At 28 dph, the larvae fed with T4 (1.687 ± 0.09 U/mg protein) showed significantly (*p* < 0.05) the highest amylase activity compared to those fed with T5 (1.382 ± 0.01 U/mg protein) and T3 (1.510 ± 0.003 U/mg protein), and the lowest activity was obtained in the larvae fed with T2 (1.147 ± 0.01 U/mg protein) and T1 (0.859 ± 0.009 U/mg protein). At 32 dph, the larvae fed with T4 (0.694 ± 0.04 U/mg protein) showed significantly (*p* < 0.05) higher amylase activity, followed by those fed T5 (0.665 ± 0.01 U/mg protein), and the lowest activity was obtained in the larvae fed T2 (0.419 ± 0.008 U/mg protein) and T1 (0.486 ± 0.04 U/mg protein).

### 3.4. Ontogeny of Protein Metabolic Enzymes in Channa striatus Larvae Fed Different Experimental Diets

**Figure 8:**
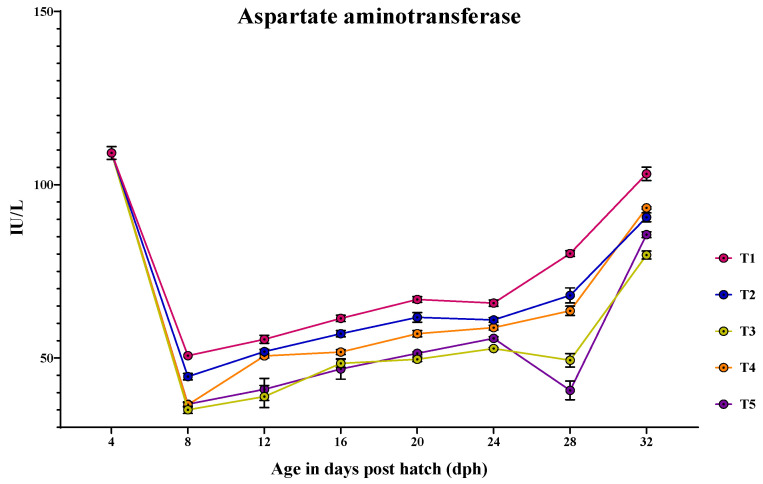
Ontogenical pattern of AST activity of *C. striatus* fed different experimental diets.

**Figure 9:**
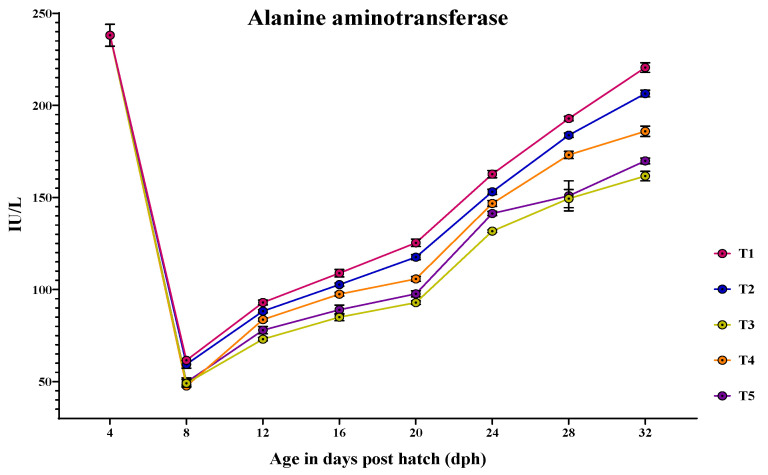
Ontogenical pattern of ALT activity of *C. striatus* fed different experimental diets.

The activity of AST and ALT showed a peak at 4 dph, followed by sudden reduction at 8 dph and increased until 32 dph (Figure 8 and Figure 9). At 4 dph, there was no significant difference (*p* > 0.05) in the AST and ALT activity among the treatments. From 8 to 32 dph, AST and ALT activity varied significantly (*p* < 0.05) among different treatments.

At 8 dph, the larvae fed with T1 (50.682 ± 1.15 U/L) showed significantly (*p* < 0.05) higher AST activity compared to other treatments. At 12 dph, the larvae fed with T1 (55.397 ± 1.15 U/L) showed the highest AST activity, which did not vary significantly (*p* > 0.05) from those feds with T2 (51.861 ± 0.64 U/L) and T4 (50.609 ± 0.58 U/L). At 16 dph, the highest AST activities were observed in larvae fed with T1 (61.438 ± 0.92 U/L); however, it was not different from T2 (57.018 ± 0.92 U/L). From 20 dph to 32 dph, the larvae fed with T1 diet showed the highest AST activity (*p* < 0.05).

For ALT activity, the larvae fed with T1 (61.585 ± 1.30 U/L) showed the higher ALT activity at 8 dph, which was not significantly different (*p* > 0.05) from those feds with T2 (59.375 ± 2.07 U/L). From 12 to 24 dph, the larvae fed with T1 showed the highest ALT activity (*p* < 0.05). At 28 dph, the larvae fed with T1 (192.859 ± 1.20 U/L) showed the higher ALT activity, which did not vary significantly from those fed with T2 (183.798 ± 1.22 U/L). At 32 dph, the larvae fed with T1 (220.558 ± 2.58 U/L) showed significantly (*p* < 0.05) higher ALT activity, followed by those fed with T2 (206.414 ± 1.84 U/L) and T4 (185.934 ± 2.81 U/L).

### 3.5. Histomorphological Development of Digestive System in Channa striatus Larvae Fed Different Experimental Diets

#### 3.5.1. Intestine

**Figure 10:**
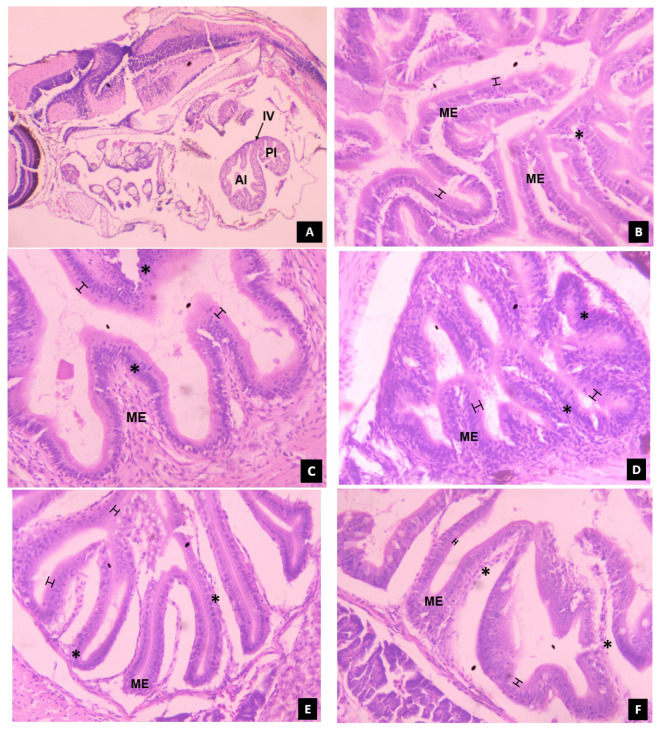
The histological development of intestine in *C. striatus* larvae fed different experimental diets. (**A**) showing the general view of anterior intestine, posterior intestine, and ileorectal valve at 4 dph (Magnification = 4×; stain—H&E); (**B**–**F**) 16 dph (Magnification = 40×; stain—H&E); (**B**) co-feed diet (T2-live feed and formulated micro diet); (**C**) protease supplemented formulated micro diet (T4); (**D**) commercial diet (T5); (**E**) live feed (T1); (**F**) formulated micro diet (T3). Abbreviations: AI, anterior intestine; PI, posterior intestine; IL, ileorectal valve; ME, mucosal epithelium; The asterisk denotes the presence of enterocytes in the mucosal epithelium; 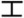, denotes the thickness of microvilli in the intestinal mucosal epithelium.

At 4 dph, the incipient intestine was well differentiated into anterior (prevalvular) and posterior (postvalvular) intestine as shown in Figure 10. The presence of the intestinal valve or ileorectal valve, which divides the two regions of the intestine and forms a constriction in the intestinal mucosa, was also observed. The intestine showed the presence of mucosal columnar epithelium with incipient microvilli and a high number of goblet cells. At 16 dph, the larvae fed with T2 showed a well-developed intestine with high folding. The enterocytes were densely packed, and a prominent layer of microvilli was present. Similar results were observed in the larvae fed with T4, except for wider and less dense intestinal folds. The larvae fed with T5 showed a shorter and dense mucosal epithelium fold with a prominent layer of microvilli, while the larvae fed with T1 showed a longer mucosal epithelium fold with indistinct microvilli. The larvae fed with T3 showed wider and shorter mucosal epithelium folds with indistinct microvilli and unevenly packed enterocytes.

#### 3.5.2. Stomach

**Figure 11:**
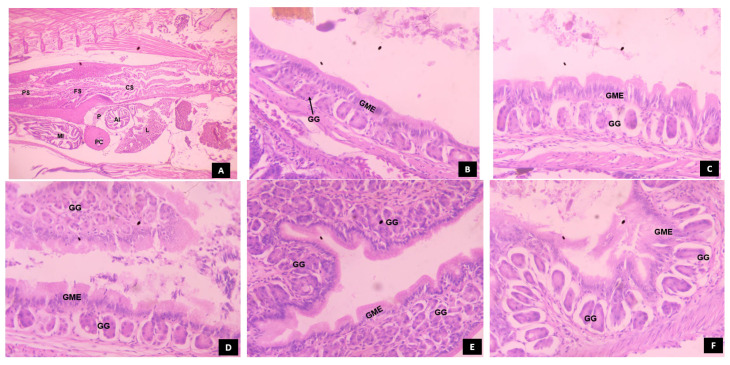
The histological development of stomach in *C. striatus* fed different experimental diets. (**A**) showing general view of digestive system and its accessory digestive glands at 12 dph (Magnification = 4×, stain—H&E); (**B**–**F**) 20 dph (Magnification = 40×, stain—H&E); (**B**) Live feed (T1); (**C**) co-feed diet (T2-live feed with formulated micro diet); (**D**) commercial diet (T5); (**E**) protease formulated micro diet (T4); (**F**) formulated micro diet (T3). Abbreviations: CS, cardiac stomach; FS, fundic stomach; PS, posterior stomach; AI, anterior stomach; MI, median intestine; PI, posterior intestine; PC, pyloric caeca; P, pancreas; L, liver; GME, gastric mucosal epithelium; GG, gastric glands.

The stomach is the last organ to differentiate in the digestive system. At 12 dph, a muscular stomach emerged from the narrow posterior part of the esophagus and got differentiated into three regions, namely cardiac stomach, fundic stomach, and pyloric stomach as shown in Figure 11. At 20 dph, the larvae fed with T4 showed densely packed, fully developed gastric glands accompanied by a thicker gastric mucosal epithelium. Inversely, the larvae fed with T3 showed loosely packed, elongated gastric glands with a thicker gastric mucosal epithelium. The larvae fed with T5 showed loosely packed, developing gastric glands with thicker gastric mucosal epithelium. However, the larvae fed with T1 and T2 showed underdeveloped and loosely packed gastric glands, with a thinner mucosal epithelium.

#### 3.5.3. Pancreas

**Figure 12:**
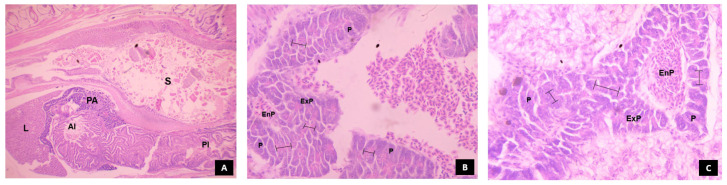

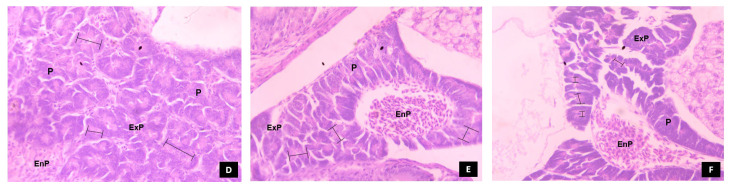
The histological development of pancreas in *C. striatus* larvae fed different experimental diets. (**A**) showing the complete development of pancreas associated with the digestive system (Magnification = 4×; stain—H&E) at 12 dph; (**B**–**F**) 12 dph (Magnification = 40×; stain—H&E); (**B**) protease-supplemented formulated micro diet (T4); (**C**) co-feed diet (T2-live feed and formulated micro diet); (**D**) formulated micro diet (T3); (**E**) commercial diet (T5); (**F**) live feed (T1). Abbreviations: AI, anterior intestine; PI, posterior intestine; S, stomach; PA, pancreas; L, liver; P, Pancreocytes; ExP, exocrine pancreas; EnP; endocrine pancreas; 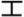, denotes the prevalence of zymogen granules in the pancreocytes arranged in acinus.

The histological development of the pancreas in *C. striatus* fed different experimental diets is shown in Figure 12. The pancreas is composed of two parts: the endocrine part and the exocrine part (pancreocytes containing zymogen granules). At 4 dph, an incipient pancreas was visible with simple cubic cells surrounded by a layer of connective tissues. At 16 dph, the larvae fed with T2 and T4 showed larger pancreocytes arranged in acini with prominent zymogen granules. The larvae fed with T3 showed densely arranged smaller pancreocytes with prominent zymogen granules compared to those larvae fed with T5. The larvae fed with T1 showed loosely arranged pancreocytes with a smaller number of zymogen granules.

#### 3.5.4. Liver

**Figure 13:**
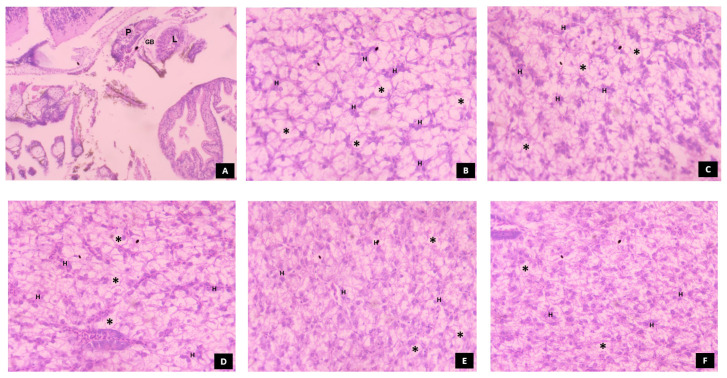
The histological development of liver in *C. striatus* fed different experimental diets. (**A**) showing presence of incipient liver below pancreas and gall bladder at 4 dph (Magnification = 40×; stain—H&E); (**B**–**F**) 12 dph (Magnification = 40×, Stain—H&E); (**B**) Live feed (T1); (**C**) co-feed diet (T2-live feed with formulated micro diet); (**D**) commercial diet (T5); (**E**) formulated micro diet (T3); (**F**) protease-supplemented formulated micro diet (T4). Abbreviations: P, pancreas; L, liver; GB, gall bladder; H, hepatocytes; The asterisk denotes the presence of lipid deposits in the liver.

At 4 dph, the incipient liver was visible as a cluster of round basophilic cells (hepatocytes) above the anterior intestine, adjacent to the liver a s shown in Figure 13. At 12 dph, the liver size had increased, with well differentiated hepatocytes and visible fat deposits in all treatment groups. The larvae fed with T1 showed the largest lipid deposits and highest number of hepatocytes, followed by those larvae fed with T2. In contrast, the larvae fed with T4 showed smaller lipid deposits but a greater number of hepatocytes compared to the other treatments. Although the larvae fed with T3 had small lipid deposits compared to those larvae fed with T5, they showed a higher number of hepatocytes.

## 4. Discussion

Fish survival and growth are influenced by several factors, such as type and composition of the diet, digestive capacities, and gut morphology development [21]. In the present study, the larvae fed with T1 showed better survival and growth performance at 8 dph compared to all other treatments. This could be due to the improved digestion and assimilation of absorbed nutrients, aided by the addition of exogenous digestive enzymes through *Artemia* nauplii. Similar findings have been reported in many fish species [21,37,38,39]. While the larvae fed with T2 showed significant growth, they failed to achieve the same level of growth and survival as the larvae fed with T1. This result is in accordance with Kolkovski [40], who stated that the growth and survival of fish larvae fed with micro diets have not matched those larvae fed with live feeds, including *Artemia* nauplii and rotifers. The low survival and growth in fish larvae fed other than T1 may be due to the poor acceptance of formulated micro diets and requires a high degree of protein degradation efficiency for effective utilization of a formulated micro diet. It also can be correlated with the underdeveloped digestive system to process the artificial micro diet at 8 dph. These results are in accordance with the findings of Civera-Cerecedo et al. [41] and Engrola et al. [42] in *Paralabrax maculatofatofasciatus* and *Senegalese sole*.

As the larvae grow, the larvae fed with T1 and T2 showed similar results in survival and growth performance (*p* > 0.05), which were higher than other treatments at 12 dph. The increased growth performance of the larvae fed with T2 may be attributed to the combined action of exogenous enzymes from *Artemia* nauplii and different protein proportions from a formulated micro diet. This formulated micro diet contains simple pre-digested proteins, including fish protein hydrolysate (FPH) and soy protein concentrate (SOC), which could aid in the digestion and absorption of formulated micro diet at 12 dph. In African catfish, larvae fed on a combination of *Artemia* nauplii and artificial micro diet also reported similar results [43]. Similarly, larvae fed with a combination of live feed and micro particulate diet containing fish protein hydrolysate improved growth performance and survival rate compared to the larvae fed solely on live feed and formulated micro diet in climbing perch [44]. Although the protease-supplemented micro diet (T4) improved the growth performance of the larvae, the larvae fed with T1 and T2 showed significantly (*p* < 0.05) higher growth performance than the larvae with T4. This differences in performance may be attributed to the variations in the quality of exogenous enzymes provided through *Artemia* nauplii and formulated micro diet during the early developmental stages, as reported by Kemigabo et al. [21]. However, the survival performance of larvae fed with T4 showed no significant difference compared to those larvae fed with T1 and T2.

In comparison to the larvae fed with T1, the larvae fed with T2 and T4 showed higher survival and growth performance at 16 dph. This may be because of the induced effect of exogenous enzymes on the secretion of endogenous enzymes during early developmental stages, when the pancreas has developed enough to secrete pancreatic enzymes as shown in Figure 12. This, in turn, results in complete digestion and/or enhanced absorption of nutrients, facilitated by improved morphological status of the intestine as shown in Figure 10. The low survival and growth performance of the larvae fed with T1 might be due to the lack of certain essential nutrients in the live feed, resulting in energy production through body nutrient reserves [45,46] which ultimately affects growth and survival rate, as observed in climbing perch [44] when feeding solely on live feed resulted in starvation.

There was no significant difference (*p* > 0.05) in the survival rate from 24 dph to 32 dph among all treatments. This might be because all diets provided sufficient nutrients to meet the larvae’s basic survival needs. The larvae fed with T4 showed higher growth performance compared to all other treatments, followed by those fed with T3 from 20 to 32 dph. These results may be attributed to the advantage of protease enzymes in improving the protein utilization capacity of fish by increasing the activity of digestive enzymes which in turn leads to advancement of digestive system development and its accessory glands at 20 dph as shown in Figure 10, Figure 11, Figure 12 and Figure 13 compared to other treatments.

At the end of the feeding trial, the cumulative mortality was higher in the larvae fed with T5 and T3, which was inversely proportional to the larvae fed with T1, followed by T4 and T2. In the present study, it was noticed that cumulative mortality is influenced by mortality during the initial exogenous feeding phase with different experimental diets. As mentioned earlier, the high cumulative mortality is due to lower feed intake and an immature digestive system that does not secrete sufficient digestive enzymes for efficient digestion and absorption of nutrients during early developmental stages. On the other hand, the low cumulative mortality is supported by exogenous enzymes provided through *Artemia* nauplii and a protease-supplemented diet, which positively affects the secretion of endogenous enzymes, development of the digestive system, and the digestion of ingested feed. These results are consistent with Saputra et al. [11], who reported that feeding live feed during early developmental stages decreased cumulative mortality in *Channa striatus* larvae compared to other treatments. Although micro diets fed with *Artemia* nauplii and protease supplementation showed low cumulative mortality, it was significantly lower than the larvae fed with T1. Similar results were reported in African catfish larvae, where Kemigabo et al. [21] found that larvae fed with live feed showed high survival, followed by a protease-supplemented diet during early developmental stages. Akbary et al. [38] reported that feeding rainbow trout larvae with a mixture of *Artemia* nauplii and an artificial diet showed significantly lower cumulative mortality compared to feeding solely on *Artemia* nauplii for a few days or a commercial starter diet.

In our investigation, the larvae fed with T4 showed significantly higher weight gain (WG), specific growth rate (SGR), and average daily growth (ADG). This might be due to enhanced protein breakdown and retention rate in the later stages of development (i.e., 16 dph to 32 dph) when all the digestive organs are fully developed as shown in Figure 10, Figure 11, Figure 12 and Figure 13 compared to the other treatments. However, studies on the effect of a protease-supplemented formulated micro diet on WG, SGR, and ADG are limited to compare the findings of the present study. The larvae fed with T1 showed significantly (*p* < 0.05) higher protein efficiency ratio (PER) and feed conversion ratio (FCR). This may be due to the better feed intake and high digestibility of *Artemia* nauplii, which has a natural composition and live enzymes. This results in efficient nutrient absorption and utilization, leading to better FCR and PER. In contrast, the FCR and PER of the larvae fed with live feed were significantly lower than those fed with a co-feed diet and formulated micro diet in African catfish larvae [47]. However, the FCR was significantly higher in rainbow trout larvae fed a commercial micro diet and *Artemia* nauplii for up to 3 days [38].

The ability of larvae to consume feed is directly related to the activity of digestive enzymes, which are crucial for nutrient breakdown, larval development, maintenance, and energy production for growth [48]. Understanding the ontogenesis of digestive enzymes is essential for interpreting the development of fish digestive and physiological capacity [15,49,50,51]. This knowledge is crucial for improving feeding protocols and ultimately enhancing the growth and survival. In this experiment, all digestive enzymes, including trypsin, chymotrypsin, total protease, amylase, and lipase, were detected at 4 dph with no significant differences (*p* > 0.05) among different treatments. This indicates that the synthesis of digestive enzymes is genetically programmed before the exogenous feeding phase [52].

After the endogenous nutritional phase, alkaline proteases such as trypsin and chymotrypsin take on the responsibility of protein digestion. These enzymes play a crucial role in supporting rapid growth during early larval development, until the functional stomach develops for acid digestion [17,53,54]. In the present study, trypsin-specific activity increased and peaked at 16 dph and consistently decreased towards 32 dph. Although larvae fed a protease-supplemented formulated micro diet showed significant activity compared to those fed with T3 and T5, larvae fed with a diet that includes *Artemia* nauplii (T1 and T2) showed higher activity at 8 and 12 dph. This could probably be due to the stimulating effects of exogenous enzymes in activating the zymogens present in the exocrine pancreas, resulting in enhanced secretion of alkaline proteases [15]. Moving towards the 16 dph of ontogenesis, larvae fed with T2 and T4 showed significantly higher activity compared to other treatments. This could be attributed to the increased secretion of larvae’s own pancreatic enzymes when they use dietary enzymes to hydrolyze the ingested proteins to expose amino acids and peptides. On the other hand, larvae fed solely with *Artemia* nauplii showed lower activity compared to those fed with T2 and T4 because the exogenous enzymes present in the live feeds are sufficient for its complete digestion, thereby not exposing any substrate for enhanced secretion of pancreatic proteases. The secretion of pancreatic enzymes is closely linked with the development of the exocrine pancreas [55]. Paray et al. [36] reported that the exocrine pancreas matured between 15 and 25 dph in *Channa striatus* larvae. When correlating the specific activity of trypsin and chymotrypsin with histological observation of the exocrine pancreas at 16 dph, it was found that an increased number of zymogen granules were noticed, along with well-developed pancreocytes arranged in an acinus in the larvae fed with T2 and T4 (as shown in Figure 12).

The subsequent reduction in pancreatic protease enzyme levels was associated with the development of gastric glands in the glandular stomach [56]. The quantification of pepsin enzyme activity has been used as a biomarker to assess the development of the functional stomach in fish larvae [51,57,58]. In the present investigation, the specific activity of pepsin was observed at 4 dph, peaking at 20 dph and remained high until 32 dph. The larvae fed with T4 showed a higher peak of pepsin-specific activity at 20 dph, coinciding with the complete development of the stomach characterized by densely arranged gastric glands (Figure 11). This could be due to the direct introduction of the exogenous enzymes from T4 into the digestive system, allowing the larvae to utilize nutrients more efficiently and resulting in faster development of the functional stomach. Conversely, the larvae fed with T1 and T2 rely on endogenous enzymes, which are not directly introduced into the digestive system, leading to slower development of gastric glands compared to the larvae fed with T4. Paray et al. [57] found that the gastric glands were well developed at 16 dph, proliferated actively, and underwent complete maturation at 20 dph in *Channa striatus* larvae. In our study, the gastric glands were completely matured at 20 dph in the larvae fed with T4. However, the studies on the effect of dietary protease on the development of the functional stomach in fish larvae are still limited. Based on the proteolytic enzymatic profile during the ontogeny of *Channa striatus*, it is evident that trypsin and chymotrypsin show complementary effects until the onset of pepsin activity in digesting ingested protein.

The activities of total protease, amylase, and lipase serve as a marker for protein, carbohydrate, and lipid metabolism [11,59]. In this study, we observed that the total protease activity increased and reached its peak at 16 dph, which was consistent with the specific activity of trypsin, chymotrypsin, and pepsin. This peak at 16 dph was due to the combined activity of intestinal proteolytic enzymes (trypsin and chymotrypsin), and then decreased towards 32 dph with the increased activity of the stomach proteolytic enzyme (pepsin) alone. This shift may be due to the transition from alkaline digestion to acid digestion. The larvae fed with T1 and T2 showed increased total protease activity at 12 dph. Live feed contains its own protease enzymes, which supports the digestive system of the larvae during the initial exogenous nutritional stage. However, feeding solely with *Artemia* nauplii at later stages may not enhance the potential of larvae’s digestive system, leading to lower protease activity at 16 dph compared to other treatments. On the other hand, larvae fed with T4 and T2 showed increased activity at the peak point at 16 dph. Generally, feeding a formulated micro diet induces the production of digestive enzymes to facilitate the digestion of the ingested feed [17,60]. Supplementing the diet with *Artemia* nauplii and protease supplementation along with the formulated micro diet further enhances the larvae’s ability to digest proteins at the early developmental stages [15]. From 20 dph, the specific activity of total protease decreased due to increased activity of acid protease, which is inversely proportional to the activity of pancreatic proteases. However, the larvae fed with T4 from 20 dph showed an increased specific activity of total protease, which could be attributed to the complete development of gastric glands or the larvae becoming more efficient in utilizing the ingested feed supplemented with proteases.

The lipase-specific activity was detected at 4 dph, peaking at 8 dph, and then decreasing towards 32 dph. The low activity at 4 dph might be due to the complete utilization of the yolk sac, which contains a high level of lipids. However, at 4 dph the larvae were not yet fully feeding on the exogenous feed. As the larvae began to actively feed on the exogenous feed, the specific activity of lipase increased to cope with the dietary lipid sources. In this regard, Martinez et al. [61] stated that the higher initial lipase-specific activity was probably due to the development of exocrine pancreas. In our study, the lipase activity followed a similar trend, and a well-developed pancreas was visible at 16 dph. In contrast, lipase-specific activity gradually increased until 15 dph after hatching and remained stable until the end of the study in *Paralichthys californicus* larvae [62]. This finding was correlated with the lipid accumulation in the liver as described by Gisbert et al. [49], which showed the capacity of lipid digestion and absorption compared to early developmental stages. However, it has been found that the specific activity of lipase was reported to be higher at early developmental stages and then decreased during development in *Lates calcarifer* [63], which could be attributed to the effect of diet quality and quantity [64]. The drastic decrease up to 32 dph could be due to maintaining the balance between enzyme production and dietary needs as the larvae approach the juvenile stage. Alternatively, this could be attributed to the utilization of lipids for tissue growth (cell membrane and energy) during early development. Once organogenesis and tissue development are complete, the demand for lipid digestion decreases leading to a reduction in lipase enzyme production.

The larvae fed with T1 showed higher lipase-specific activity, followed by T2 and T4 at 8 dph. This could be attributed to the presence of easily digestible lipids, particularly polyunsaturated fatty acids (PUFAs), in *Artemia* nauplii. These PUFAs stimulate the production of endogenous pancreatic lipase secretion in the developing larvae, as efficient digestion is necessary for absorption, as described by Kolkovski et al. [65] and Kamaszewski et al. [66]. Subsequently, the larvae fed with T2 showed no significant difference in the lipase-specific activity compared to the larvae fed with T1 at 12 dph. The larvae fed with T4 showed lower lipase-specific activity but significantly higher than the larvae fed with T3 and T5. This indicates that their pancreas is developing to digest the less readily digestible lipids available from fish oil, sunflower oil, and soy lecithin. As mentioned earlier, the exocrine pancreas was well developed at 16 dph in the larvae fed with T4 and T2, with no significant difference between them. Consistent with this result, the lipase-specific activity was higher in the larvae fed with T4 and T2 at 20 dph. This shows that the exocrine pancreas had developed enough to secrete more pancreatic lipase to digest the dietary lipids from fish oil, sunflower oil, and soy lecithin with the assistance of exogenous enzymes. Previous studies have shown that dietary protease increases lipase activity as reported in Juvenile sterlet [67,68].

In the ontogenical pattern of amylase during the early developmental stages, the specific activity of amylase showed a peak at 4 dph, followed by a drastic reduction with another peak at 28 dph in all treatments. This initial peak is likely due to the transition of larvae’s digestive system from endogenous yolk nutrients to external feeding, resulting in the release of various enzymes, including proteases, amylase, and lipase. Pancreatic enzymes have a non-specific response to the food, causing their release. Hamza et al. [69] found similar effects, reporting that the specific activity of amylase initially peaked at first feeding and then sharply decreased in pikeperch larvae. The significant decrease in amylase specific activity may be because larvae prefer proteins and fats over carbohydrates during early developmental stages. In the present study, larvae fed with T1 showed significantly higher activity up to 12 dph compared to other treatments, which may be due to stimulatory effects of *Artemia* nauplii in secreting endogenous pancreatic amylase. Saputra et al. [11] also observed the same effects in *Channa striatus* larvae, where amylase activity peaked initially when fed with live feed. At 16 dph, larvae fed with T4 and T2 showed significantly higher amylase activity followed by larvae fed with T1, coinciding with the complete development of the pancreas with more zymogen granules (Figure 12). From 20 dph, larvae fed with T4 began to dominate in specific activity of amylase possibly due to a combination of enhanced amylase activity and a well-developed digestive system responsible for carbohydrate digestion. Lin et al. [70] reported that supplementing protease enzymes at a rate of 1.5 g/kg increased amylase activity in Nile tilapia. However, amylase specific activity varies according to species, stages, and dietary factors [29].

The protein metabolic enzymes aspartate aminotransaminase (AST) and alanine transaminase (ALT) are indicators of protein metabolism, growth condition, and stress levels in fish [44,71]. In this study, both AST and ALT showed peak activity soon after absorbing the yolk sac but decreased instantly at 8 dph and increased until the end of the study. At 4 dph, the initial high activity of AST and ALT indicates active metabolism related to yolk sac absorption, as these enzymes are involved in liver function and amino acid metabolism. The decreased activity at 8 dph may be due to the transition from endogenous to exogenous feeding, where the larvae were fed a different diet. The subsequent increase in activity likely reflects the stress or larval growth. The higher activity of AST and ALT can be explained in two ways in relation to growth and stress levels, as they correlate with fat accumulation in the liver. In the present study, larvae fed with T1 showed higher AST and ALT activity, as well as higher growth and large fat deposits at 12 dph. Therefore, the increased activities of AST and ALT, along with the presence of large fat deposits, indicate that *Artemia* nauplii have a low total lipid content with a low proportion of phospholipid content [23]. This lack of phospholipids hinders the transportation of absorbed lipids, causing stress to the liver.

Although feeding *Artemia* nauplii causes liver stress, it improved the growth performance at 12 dph, indicating the participation of apparently produced non-essential amino acids in cellular metabolism [44]. However, this condition did not remain stable until the end of the study. The growth of larvae fed solely with T1 showed decreased growth performance after 12 dph compared to other treatments. This is because the live feed lacks certain essential amino acids, which are required for adequate growth [46]. In contrast, larvae fed with T4 showed higher activities of AST and ALT, but smaller fat deposits (Figure 13), as well as higher growth performance, although significantly lower than larvae fed with T1 and T2 at 12 dph. This might be due to enhanced lipase activity through exogenous enzyme action and higher proportion of phospholipids present in the protease-supplemented formulated micro diet containing soy lecithin [72,73]. These factors aid in enhanced lipid digestion, absorption, and transportation. The increased activities of AST and ALT, along with high growth, may be due to less stress and the participation of synthesized amino acids in supporting growth performance, as described by Prakash et al. [44].

## 5. Conclusions

In this study, it is clearly shown that growth and survival were the main challenges during the early phase of larval rearing, particularly when the digestive system is developing. The research findings show that different weaning diets had significantly influenced the survival, growth, and ontogeny of the digestive system. In the present study, the larvae fed with *Artemia* nauplii (T1) showed the best performance in terms of survival, growth, and enzyme activity compared to other diets, but relying solely on *Artemia* nauplii is not feasible due to its limitations in nutrient content and high production costs, which necessitates an alternative approach. Conversely, co-feeding with *Artemia* nauplii and a formulated micro diet (T2) effectively supported the larval performance at 12 dph similar to the larvae fed with T1. This signifies that weaning of *Artemia* fed *C. striatus* larvae with co-feed diet (*Artemia* nauplii and formulated diet) can be carried out from 9 dph. The larvae fed with a protease-supplemented micro diet (T4) showed comparable performance to those fed with T2 diet at 16 dph and showed significantly better performance from 20 dph. This suggests that the co-feed diet fed to *C. striatus* larvae can be transitioned to a protease-supplemented micro diet (T4) from 17 dph. These findings suggest a weaning protocol based on the biochemical and histological aspects of digestive system development in *C. striatus* larvae. This study provides an insight for developing age specific feed and feeding regimes for effective larval rearing of *C. striatus*. This study also emphasizes the importance of dietary protease supplementation during early weaning, highlighting the need to evaluate its stability in water and its effectiveness in the larval gut to enhance nutrient utilization during early developmental stages.

## Figures and Tables

**Table 1:** Experimental Design.

Test Diet	Weaning Method
T1	Live feed (*Artemia* nauplii instar stage I)
T2	Live feed + Formulated micro diet
T3	Formulated Micro diet
T4	Formulated Micro diet with protease enzyme supplementation
T5	Commercial larval diet

**Table 2:** Ingredient composition and nutrient content of the formulated micro diet (dry matter basis).

Ingredients (% of Dry Weight)	Formulated Micro Diet	Formulated Diet with Protease Enzyme
Tuna Fish Meal	44	44
Squid meal	11	11
Fish protein hydrolysate	10	10
Shrimp heal meal	5	5
Soy protein concentrate	8.3	8.3
Wheat flour	8	7.9
Sunflower oil	2.5	2.5
Fish oil	2.5	2.5
Soy lecithin	2	2
Vitamin premix ^1^	1.5	1.5
Mineral premix ^2^	1.5	1.5
Vitamin C	1.5	1.5
Vitamin E	0.2	0.2
L-Tryptophan	0.5	0.5
Carboxy methyl cellulose	1.5	1.5
Protease enzyme ^3^	-	0.1
Proximate Composition (%) ^4^		
Moisture	10.34	10.41
Crude protein	52.43	52.26
Crude lipid	12.44	12.21
Total Ash	15.34	15.54

^1^ The vitamin premix provided the following per kilogram of the diet: Vit. A—10,000,000 IU, Vit. B1—5000 mg, Vit. B2—5000 mg, Vit. B3—6000 mg, Vit. B5—6000 mg, Vit. B6—6000 mg, Vit. C—60,000 mg, Vit. D3—2,000,000 IU, Vit. E—10,000 IU, Vit. H—200 mg. ^2^ The mineral premix provided the following per kilogram of the diet: Magnesium—2800 mg, Iodine—7.4 mg, Iron—7400 mg, Copper—1200 mg, Manganese—11,600 mg, Zinc—9800 mg, Chlorides cobalt—4 mg, Potassium—100 mg, Selenium—4 mg, Calcium carbonate—27.25%, Phosphorous—7.45 mg, Sulphur—0.7 mg, Sodium—6 mg, Calpan—200 mg, Aluminium—1500 mg and Choline chloride—10,000 mg. ^3^ Biovencer Healthcare Pvt. Ltd., Greater Noida, Uttar Pradesh, India. ^4^ Analyzed according to the procedures followed by standard AOAC [30].

**Table 3:** Proximate composition of *Artemia* nauplii instar I and Commercial feed (dry weight basis).

Parameters (%) ^1^	*Artemia* nauplii Instar I	Commercial Diet
Moisture	84.14	12.3
Crude protein	53.29	52.67
Crude lipid	15.23	12.23
Total ash	8.89	12.8

^1^ Analyzed according to standard procedure of AOAC [30].

**Table 4:** Survival and cumulative mortality rate of *Channa striatus larvae* fed different experimental diets.

Parameter	Treatments					*p*-Value
	T1	T2	T3	T4	T5	
Survival rate (%)						
8-days post hatch	80.479 ± 1.71 ^a^	61.979 ± 0.62 ^c^	65.688 ± 1.65 ^bc^	62.854 ± 1.38 ^c^	68.562 ± 0.53 ^b^	<0.001
12-days post hatch	93.113 ± 0.35 ^a^	90.665 ± 1.19 ^a^	83.280 ± 1.49 ^b^	90.627 ± 2.67 ^a^	83.896 ± 0.38 ^b^	0.003
16-days post hatch	87.924 ± 0.24 ^bc^	92.414 ± 1.89 ^ab^	94.206 ± 2.47 ^a^	96.973 ± 0.33 ^a^	86.328 ± 1.88 ^c^	0.005
20-days post hatch	98.858 ± 0.25 ^a^	99.858 ± 0.14 ^a^	99.329 ± 0.26 ^a^	99.580 ± 0.34 ^a^	94.326 ± 1.77 ^b^	0.004
24-days post hatch	99.256 ± 0.38 ^a^	99.812 ± 0.12 ^a^	99.770 ± 0.164 ^a^	99.899 ± 0.05 ^a^	98.042 ± 1.17 ^a^	0.183
28-days post hatch	99.964 ± 0.035 ^a^	99.558 ± 0.15 ^ab^	99.858 ± 0.08 ^a^	99.362 ± 0.56 ^ab^	98.432 ± 0.54 ^b^	0.081
32-days post hatch	100.00 ± 0.00 ^a^	99.797 ± 0.20 ^a^	99.858 ± 0.08 ^a^	100.00 ± 0.00 ^a^	100.00 ± 0.00 ^a^	0.461
CM (%)	34.875 ± 1.65 ^c^	47.292 ± 0.47 ^ab^	48.313 ± 1.04 ^a^	43.208 ± 1.63 ^b^	51.708 ± 2.01 ^a^	<0.001

Note: Values are expressed as mean ± SE of three replicate per diet and values with different superscripts in the same row indicate significant differences determined by Duncan’s test (*p* < 0.05). ^1^ CM, Cumulative mortality.

**Table 5:** Growth performance of *Channa striatus* larvae fed different experimental diets.

Parameter	Treatments					*p*-Value
	T1	T2	T3	T4	T5	
^1^ IMW (mg)	0.646 ± 0.01	0.646 ± 0.01	0.646 ± 0.01	0.646 ± 0.01	0.646 ± 0.01	1.000
8-day old weight (mg)	24.896 ± 0.14 ^a^	23.020 ± 0.17 ^b^	17.863 ± 0.29 ^d^	21.016 ± 0.20 ^c^	17.896 ± 0.11 ^d^	<0.001
12-day old weight (mg)	47.136 ± 0.20 ^a^	47.110 ± 0.28 ^a^	32.813 ± 0.42 ^c^	42.066 ± 0.145 ^b^	32.833 ± 0.18 ^c^	<0.001
16-day old weight (mg)	68.533 ± 0.38 ^b^	71.620 ± 0.40 ^a^	65.023 ± 0.15 ^c^	71.600 ± 0.40 ^a^	64.556 ± 0.37 ^c^	<0.001
20-day old weight (mg)	115.890 ± 0.16 ^d^	118.966 ± 0.17 ^c^	122.023 ± 0.15 ^b^	128.156 ± 0.18 ^a^	121.890 ± 0.15 ^b^	<0.001
24-day old weight (mg)	134.856 ± 0.14 ^d^	142.103 ± 0.41 ^c^	149.566 ± 0.50 ^b^	152.356 ± 0.36 ^a^	149.086 ± 0.50 ^b^	<0.001
28-day old weight (mg)	160.240 ± 1.35 ^d^	171.066 ± 1.82 ^c^	177.526 ± 0.40 ^b^	182.426 ± 0.51 ^a^	177.626 ± 0.43 ^b^	<0.001
32-day old weight (mg)	194.606 ± 0.51 ^d^	216.123 ± 0.23 ^c^	237.047 ± 0.17 ^b^	245.870 ± 0.20 ^a^	236.240 ± 0.52 ^b^	<0.001
^2^ ADG (mg)	6.688 ± 0.01 ^d^	7.430 ± 0.008 ^c^	8.151 ± 0.006 ^b^	8.456 ± 0.007 ^a^	8.124 ± 0.17 ^b^	<0.001
^3^ MWG (mg)	193.966 ± 0.51 ^d^	215.483 ± 0.23 ^c^	236.407 ± 0.17 ^b^	245.230 ± 0.20 ^a^	235.607 ± 0.52 ^b^	<0.001
^4^ FCR	1.889 ± 0.17 ^a^	3.529 ± 0.03 ^b^	5.347 ± 0.20 ^d^	4.53 ± 0.03 ^c^	5.658 ± 0.27 ^d^	<0.001
^5^ PER	1.015 ± 0.08 ^a^	0.534 ± 0.004 ^b^	0.353 ± 0.01 ^c^	0.416 ± 0.002 ^bc^	0.341 ± 0.01 ^c^	<0.001
^6^ SGR (%/day)	19.609 ± 0.05 ^d^	19.918 ± 0.003 ^c^	20.236 ± 0.002 ^b^	20.363 ± 0.002 ^a^	20.225 ± 0.007 ^b^	<0.001

Note: Values are expressed as mean ± SE of three replicate per diet and values with different superscripts in the same row indicate significant differences determined by Duncan’s test (*p* < 0.05). ^1^ IMW, Initial mean weight; ^2^ ADG, Average daily growth; ^3^ MWG, Mean weight gain; ^4^ FCR, Feed conversion ratio; ^5^ PER, Protein efficiency ratio; ^6^ SGR, Specific growth rate.

## Data Availability

The data that support the findings of this study are available on request from the corresponding author. The data are not publicly available due to privacy or ethics.
